# Managing malignant recurrent pericardial effusion in a palliative patient with bleomycin sclerotherapy: A case report from Bangladesh

**DOI:** 10.1097/MD.0000000000050509

**Published:** 2026-09-11

**Authors:** Subrata Das, A.K.M. Motiur Rahman Bhuiyan, Afroja Alam, Chayan Kumar Singha, Mostofa Kamal Chowdhury

**Affiliations:** a Department of Palliative Medicine, Bangladesh Medical University, Dhaka, Bangladesh; b Department of Cardiology, Bangladesh Medical University, Dhaka, Bangladesh.

**Keywords:** Bangladesh, bleomycin, cardiac tamponade, lung cancer, malignant pericardial effusion, palliative care, pericardiodesis

## Abstract

**Rationale:**

Malignant pericardial effusion (MPE) is a life-threatening complication of advanced malignancies that frequently causes cardiac tamponade and severe functional decline. While pericardiocentesis provides acute relief, recurrence rates remain high. Intrapericardial sclerotherapy offers a minimally invasive approach to prevent reaccumulation and improve quality of life in palliative patients. Here, we report a case of recurrent MPE secondary to lung cancer successfully managed with intrapericardial bleomycin (BLM) pericardiodesis.

**Patient concerns:**

A 50-year-old male chronic smoker with advanced lung cancer and pericardial metastasis presented with progressive dyspnea, pleuritic chest pain, and cough due to recurrent cardiac tamponade. Initial emergency pericardiocentesis provided temporary palliation, but the effusion recurred within 2 months.

**Diagnoses:**

Echocardiography and chest imaging confirmed recurrent pericardial effusion with hemodynamically significant tamponade physiology. Cytological analysis of pericardial fluid revealed metastatic carcinoma consistent with lung cancer origin.

**Interventions:**

Emergency pericardiocentesis was performed, draining approximately 1000 ml of hemorrhagic fluid. Following confirmation of minimal residual effusion on repeat imaging, chemical pericardiodesis was performed via intrapericardial instillation of 10 mg BLM diluted in normal saline through a pigtail catheter, with the catheter clamped for 48 hours.

**Outcomes:**

The procedure was well tolerated without adverse effects. Follow-up echocardiography confirmed complete resolution of pericardial effusion. The patient experienced substantial symptomatic improvement in breathlessness, chest pain, and overall comfort, with no evidence of recurrence on subsequent follow-up.

**Lessons:**

This case underscores the safety and efficacy of intrapericardial BLM as a palliative intervention for recurrent MPE. It demonstrates the value of minimally invasive chemical pericardiodesis in preventing recurrence, enhancing quality of life, and avoiding the burden of repeated procedures; particularly relevant in resource-limited settings.

## 1. Introduction

Malignant pericardial effusion (MPE) is a well-recognized complication of advanced malignancies, most commonly associated with lung and breast cancers.^[1]^ Among the initial clinical manifestations, cardiac tamponade and concurrent pleural effusion are frequently observed. MPE is typically associated with a poor prognosis, with reported median survival of < 4 months.^[2]^

Management of MPE depends on the primary cancer type. Systemic therapy is preferred for chemosensitive tumors, while local interventions may be more effective for less responsive malignancies.^[3]^ Common procedures (such as pericardiocentesis, pericardial window, or pericardiotomy) remain controversial due to recurrence rates and procedural risks. Adjunctive intrapericardial therapies, including sclerosing or cytotoxic agents, are increasingly used to improve outcomes.^[4,5]^ Both systemic and local therapeutic strategies (used either alone or in combination) have been employed with varying degrees of success.^[6]^ Despite decades of clinical experience, a consensus regarding the optimal local therapy for MPE has yet to be established.

Several agents have been used for intrapericardial instillation, including bleomycin (BLM), doxycycline, 32P-colloid, carboplatin, cisplatin, and thiotepa, either for their sclerosing or cytotoxic properties. A comparative study of BLM and doxycycline involving 27 patients demonstrated equivalent efficacy between the 2 agents, though BLM was associated with a more favorable toxicity profile.^[7]^ Furthermore, a randomized controlled trial by Kunitoh et al^[8]^ compared BLM instillation following pericardial drainage to drainage alone and demonstrated good tolerability and improved outcomes with BLM.

In light of existing evidence and institutional experience, our center has adopted intrapericardial BLM instillation as the standard post-drainage treatment for MPE. This case report presents the clinical course of a 50-year-old male with lung cancer and pericardial metastasis who developed recurrent cardiac tamponade. Following repeated pericardial drainage, chemical pericardiodesis was performed using BLM, resulting in complete resolution of the effusion and significant symptomatic improvement. The report highlights the feasibility, safety, and clinical utility of BLM as a local therapeutic agent in managing MPE secondary to lung cancer.

## 2. Case report

A 50-year-old male, chronic smoker, nondiabetic and normotensive, presented to Bangladesh Medical University in late September 2024 with complaints of progressive shortness of breath, pleuritic chest pain, and nonproductive cough for the past 7 days. He reported being in good health approximately 8 months prior, after which he developed intermittent fever and cough. Initial treatment by a local physician with unspecified medications led to only partial symptom relief. Subsequently, he sought consultation in Dhaka, where a pulmonologist ordered further investigations.

A chest X-ray and fine-needle aspiration cytology of the left cervical lymph node revealed metastatic poorly differentiated carcinoma, suggestive of a primary malignancy in the lung or head-and-neck region. Spiral computed tomography scan of the chest demonstrated left-sided pulmonary consolidation with nodules and moderate pericardial effusion. Due to financial limitations, the patient was unable to complete further diagnostic workup and returned home.

**Figure 1.**
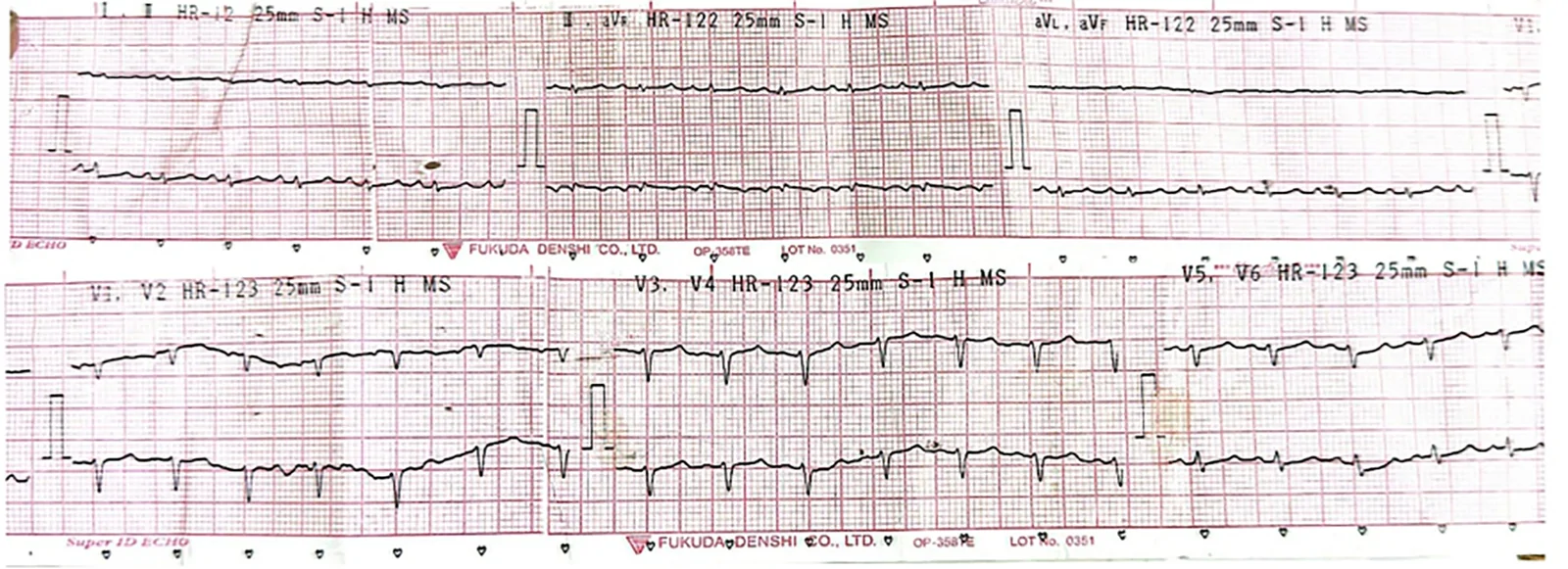
ECG of the patient before the procedure. ECG = electrocardiogram.

Within a short period, his symptoms worsened, including severe dyspnea and chest discomfort. He was admitted to a divisional medical college hospital, where electrocardiogram showed low voltage (Fig. 1) and echocardiographic findings were consistent with pericardial tamponade. Emergency pericardiocentesis was performed, yielding approximately 1200 ml of hemorrhagic fluid. Fluid analysis revealed: reddish appearance, numerous red blood cells, white blood cell count of 200/cumm with 90% lymphocytes, negative Ziehl–Neelsen and gram staining, adenosine deaminase level of 63.9 U/L, and cytological findings suggestive of metastatic carcinoma. Although his dyspnea and chest pain improved following drainage, his cough persisted.

Approximately 2 months later, the patient experienced a recurrence of his previous symptoms and was referred to the Department of Palliative Medicine, Bangladesh Medical University for further management. Upon admission, the patient underwent a comprehensive evaluation, including clinical history, physical examination, and baseline investigations. Two days post-admission, he developed worsening dyspnea and profound weakness. Emergency bedside echocardiography confirmed reaccumulation of pericardial effusion with tamponade physiology. He was promptly referred to the Department of Cardiology, where a pigtail catheter was inserted via a subxiphoid approach under aseptic precautions, draining approximately 1000 ml of hemorrhagic fluid. Intermittent drainage of 100 to 200 ml was performed on alternate days.

**Figure 2.**
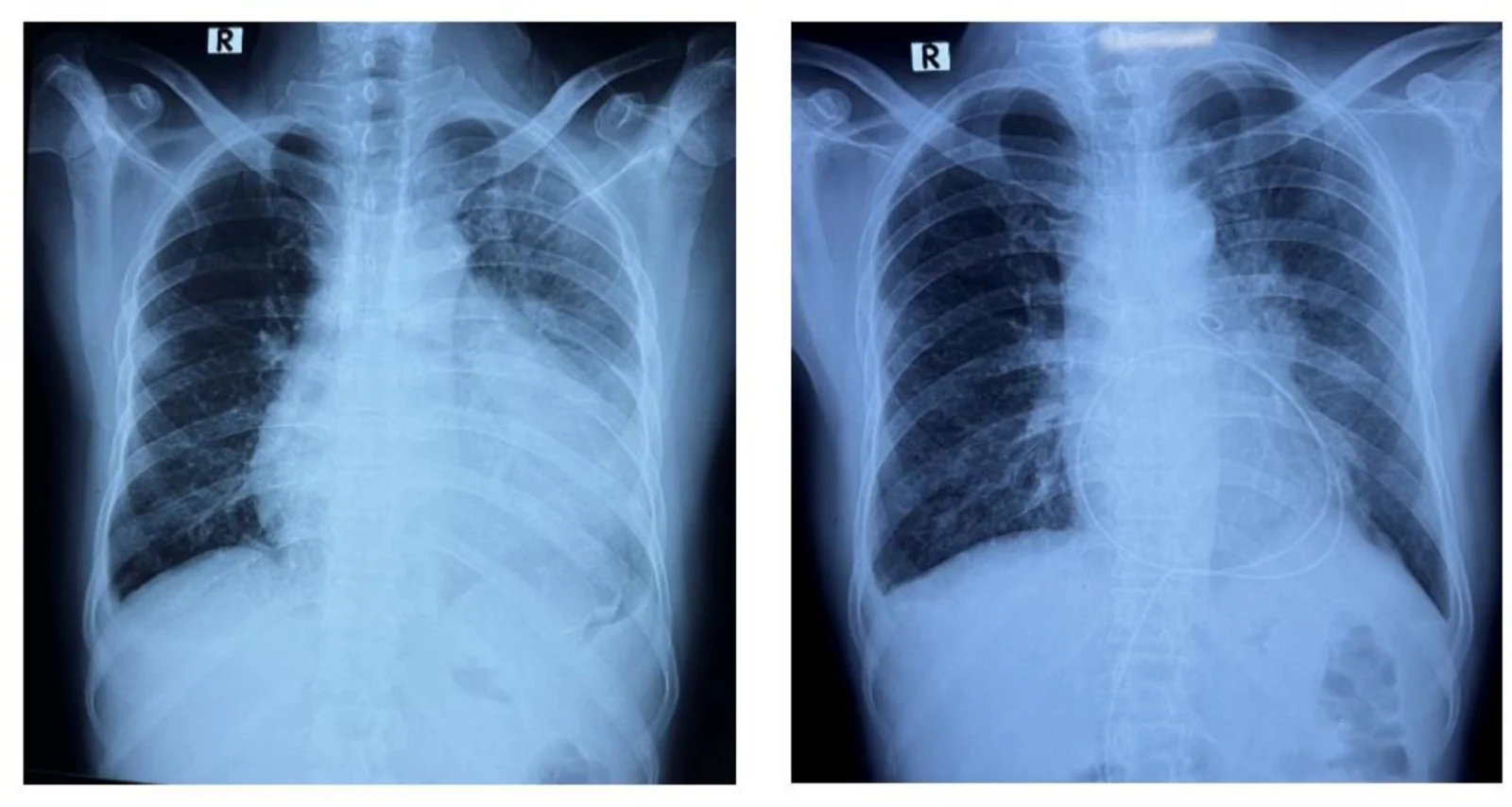
Chest X-ray P/A view before and after the procedure. P/A = posteroanterior.

Given the recurrent nature of the effusion and the underlying malignancy, when the follow-up chest radiograph (Fig. 2) and echocardiography revealed minimal residual pericardial effusion, the clinical team opted for chemical pericardiodesis using intrapericardial BLM to improve quality of life and prevent recurrence. After obtaining both verbal and written informed consent, 10 ml of 2% lignocaine was instilled into the pericardial space via the pigtail catheter, followed by 10 mg of BLM diluted in 25 ml of normal saline. The catheter was then clamped for 48 hours. The procedure was uneventful, and the patient remained hemodynamically stable during the peri-procedural period.

**Figure 3.**
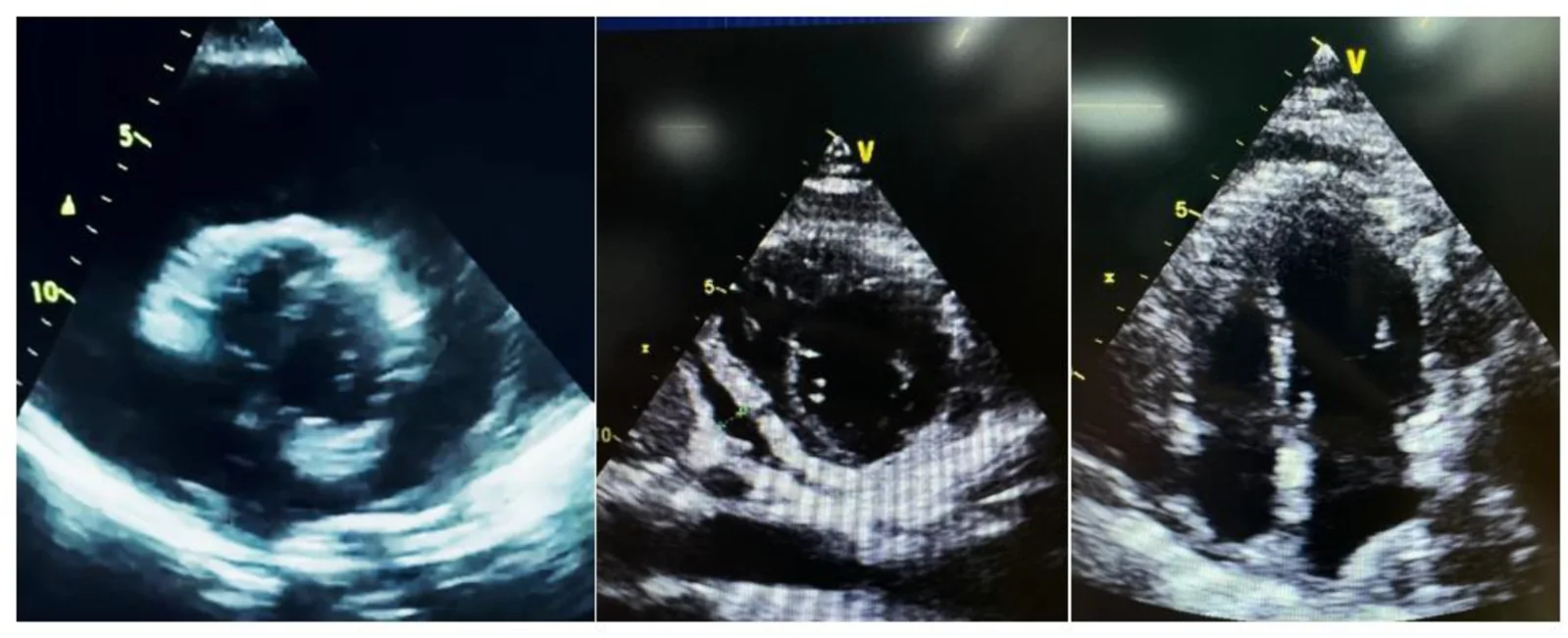
Echocardiography before and after the procedure.

At 48-hour follow-up, echocardiography (Fig. 3) showed no residual effusion. The catheter was subsequently removed without complication. The patient was monitored using the Subjective, Objective, Assessment, and Plan framework and demonstrated marked symptomatic improvement. He was discharged on request 3 days later, with instructions to return for follow-up in 15 days.

## 3. Discussion

MPE, particularly when accompanied by cardiac tamponade, represents a critical oncologic emergency that demands prompt diagnosis and intervention. Cardiac tamponade results from elevated intrapericardial pressure that compromises the filling of cardiac chambers (most notably the right atrium and right ventricle) thereby limiting venous return and ultimately reducing cardiac output.^[9]^

Management goals for MPE depend on the patient’s overall prognosis and performance status. In patients with a projected survival of several months or more, the primary objectives are symptom relief, prevention of recurrence, and improvement in quality of life, potentially extending survival through treatment of the underlying malignancy.^[10,11]^ In contrast, in terminally ill patients with limited life expectancy, the focus may be solely on symptom palliation and maintaining comfort.^[12]^

Urgent pericardiocentesis remains the cornerstone of initial management for cardiac tamponade. However, recurrence rates following simple drainage can reach up to 40%,^[1]^ indicating the need for adjunctive therapies. Although systemic chemotherapy may contribute to effusion control in chemosensitive malignancies, it is often insufficient when used in isolation, particularly in tumors that are poorly responsive to systemic agents.

Multiple strategies have been explored to prevent reaccumulation of pericardial fluid, including repeated pericardiocentesis, creation of a pericardial window, intrapericardial instillation of sclerosing agents, and both systemic and local chemotherapy.^[12,13]^ Among these, chemical pericardiodesis using cytotoxic or sclerosing agents has emerged as a minimally invasive and effective option for recurrent MPE. Studies have demonstrated that combining pericardiocentesis with local chemotherapy may offer outcomes comparable to more invasive procedures such as pericardiotomy or surgical window formation, but with fewer associated complications.^[1]^

Various intrapericardial agents have been evaluated, including BLM, thiotepa, tetracyclines, and platinum-based compounds. BLM and thiotepa, which possess both cytotoxic and sclerosing properties, have shown efficacy in reducing recurrence with relatively low toxicity, particularly in breast and lung cancer-related effusions.^[12]^ In our patient, after confirming minimal residual effusion, injection 2% lignocaine was administered intrapericardially via a pigtail catheter for local anesthesia, followed by BLM administration. The catheter was clamped for 48 hours, and the procedure was well tolerated, with stable hemodynamic parameters throughout. Subsequent imaging showed no reaccumulation of fluid, and the patient’s symptoms improved significantly.

This case highlights the utility of intrapericardial BLM as a safe and effective method for managing recurrent MPE. It underscores the importance of integrating local chemotherapy with drainage procedures, particularly in resource-limited settings where more invasive surgical options may not be feasible. The favorable outcome observed in this patient adds to the growing body of evidence supporting the use of BLM for chemical pericardiodesis in malignancy-related effusions.

## 4. Conclusion

MPE is one of the frequently encountered causes of cardiac tamponade, where the recurrence is high. Pericardiocentesis is a life-saving measure, and pericardiodesis is one of the choices to prevent recurrence. In our palliative patient, we used BLM as a sclerosing agent, and it appears to be a safe and effective way to avoid recurrence of MPE, further improving quality of life.

## Acknowledgments

The authors are grateful to the patient and her family for their cooperation and consent and to the palliative care team of Bangladesh Medical University for their support in patient management and data collection. Generative AI assistance (ChatGPT v4.0) was employed solely for text polishing under human oversight; all substantive content and interpretation remain the responsibility of the authors.

## Author contributions

**Conceptualization:** Subrata Das, A.K.M. Motiur Rahman Bhuiyan, Mostofa Kamal Chowdhury.

**Data curation:** Subrata Das, Afroja Alam.

**Formal analysis:** Subrata Das.

**Investigation:** Subrata Das, Chayan Kumar Singha.

**Methodology:** Subrata Das, Mostofa Kamal Chowdhury.

**Project administration:** Subrata Das, Chayan Kumar Singha, Mostofa Kamal Chowdhury.

**Resources:** Afroja Alam.

**Supervision:** A.K.M. Motiur Rahman Bhuiyan, Afroja Alam, Mostofa Kamal Chowdhury.

**Validation:** A.K.M. Motiur Rahman Bhuiyan, Mostofa Kamal Chowdhury.

**Visualization:** Mostofa Kamal Chowdhury.

**Writing – original draft:** Subrata Das, Afroja Alam, Mostofa Kamal Chowdhury.

**Writing – review & editing:** Subrata Das, A.K.M. Motiur Rahman Bhuiyan, Chayan Kumar Singha, Mostofa Kamal Chowdhury.
